# Good to excellent reliability of instrumented ankle passive resistance and strength measurements in children

**DOI:** 10.3389/fped.2026.1832116

**Published:** 2026-05-28

**Authors:** Alexandra Åhblom, Zhihao Duan, Antea Destro, Eva Pontén, Cecilia Lidbeck, Ruoli Wang

**Affiliations:** 1 Department of Women’s and Children’s Health, Division of Pediatric Neurology, Karolinska Institutet, Stockholm, Sweden; 2 Theme Women’s Health and Allied Health Professionals, Medical Unit Allied Health Professional, Karolinska University Hospital, Stockholm, Sweden; 3 Department of Engineering Mechanics, KTH MoveAbility, Royal Institute of Technology, Stockholm, Sweden; 4 Department of Pediatric Orthopedic Surgery and Pediatric Neurology, Astrid Lindgren Children’s Hospital, Karolinska University Hospital, Stockholm, Sweden

**Keywords:** adolescent and children, lower extremity, muscle spasticity, muscle strength, reproducibility of results, stiffness, test-retest reliability

## Abstract

**Introduction:**

Neuromuscular development is of importance for motor performance. The aim of this study was to evaluate the reliability of instrumented measurements of ankle resistance during passive movements and muscle strength in the lower leg in children.

**Methods:**

A test-retest design was used with a 2-way mixed-effects model to calculate intraclass correlation coefficients (ICC). Eleven typically developing children (eight girls), aged 7–14 years, participated. Passive ankle resistance was assessed with the NeuroFlexor, which performed controlled passive movements over 40° from plantarflexion to maximal dorsiflexion at slow and fast velocities. A biomechanical model decomposed measured passive ankle resistance (torque) into non-neural (stiffness coefficient) and maximal neural torque (neural resistance) components. Muscle strength was measured using a rig-fixed dynamometer during maximal isometric voluntary contractions of the plantarflexors and dorsiflexors. Testing was performed twice throughout the same day with a 2-hour interval.

**Results:**

ICC (95% CI) between the two measurements were as follows: stiffness coefficient: 0.79 (0.13,0.94); maximal neural torque: 0.96 (0.86,0.99); maximal voluntary contraction of plantarflexors: 0.96 (0.86, 0.99); and dorsiflexors 0.96 (0.86, 0.99).

**Conclusion:**

The NeuroFlexor and rig-fixed dynamometer showed good to excellent test-retest reliability for assessing passive ankle joint resistance and muscle strength in typically developing children. Further research is needed to explore their use in clinical populations.

## 1 Introduction

Understanding motor function in children requires reliable assessment tools for measuring passive resistance of muscles and voluntary muscle strength—two key parameters essential for evaluating neuromuscular development and identifying deviations from typical motor performance (1–3).

Passive joint resistance reflects both mechanical and neural properties and contributes to joint behavior by providing intrinsic stiffness from non-contractile tissues. This stiffness may influence limb mobility and postural stability (2, 4). To date, assessment of passive joint resistance primarily relies on subjective evaluation of resistance during manual passive extension of the examined limb. These methods are associated with questionable measurement reliability (5, 6). To improve objectivity, motorized robotic instruments such as the NeuroFlexor (Aggero MedTech AB, Älta, Stockholm, Sweden) have been developed to quantify passive resistance during controlled passive joint movements (7). Recent biomechanical approaches combining motor-driven joint rotations with torque and electromyographic measurements have further advanced mechanistic understanding of passive joint resistance. However, their complexity currently limits clinical application (41). The NeuroFlexor is designed to provide objective quantification of the relative contribution of neural and non-neural (stiffness and viscoelastic) factors to resistance during passive joint movements, offering insights into hyper-resistance.

Hyper-resistance can be defined as impaired neuromuscular response to passive muscle stretch (2). By integrating controlled passive movement with biomechanical modelling, motorized devices can distinguish neural and non-neural contributors to neuromuscular resistance (2, 8). The NeuroFlexor foot module has demonstrated high validity and intra-rater reliability in quantifying components of resistance in adults with chronic stroke (9). Similar approaches have also been applied to the upper limb, yielding good reliability (10). However, the diagnostic reliability of these motorized devices for measuring muscle resistance remains underexplored in children. Together, assessment of passive muscle resistance and voluntary muscle strength provides complementary information on neuromuscular function.

Muscle strength plays a crucial role in motor function. It enables movement, stabilizes joints, and supports functional activities such as walking. For example, forward propulsion during gait is primarily generated by the ankle plantarflexor muscles (11, 12). Effective locomotion requires both muscle force and neuromuscular coordination (13). Measuring strength in children is challenging. While muscle strength can also be assessed using isokinetic dynamometry or weight-bearing force plate protocols, clinical practice most often relies on isometric measurements, muscle contractions without changing length, using hand-held dynamometers (HHD), due to their feasibility, short testing time and suitability for pediatric clinical settings (3, 14). Standardizing joint positions is essential for obtaining valid measurements. However, this can be challenging in growing children because of ongoing changes in body proportions and motor control. Given these challenges, selecting appropriate tools for assessing muscle strength in children is critical (15). Although several studies have reported normative isometric muscle strength values at the ankle joint using an HHD (14, 16), these assessments likely provide only an estimate of true strength capacity (16, 17). While HHD is generally reliable, its accuracy can be compromised by variations in examiner-applied counterforce (18, 19).

**Figure 1:**
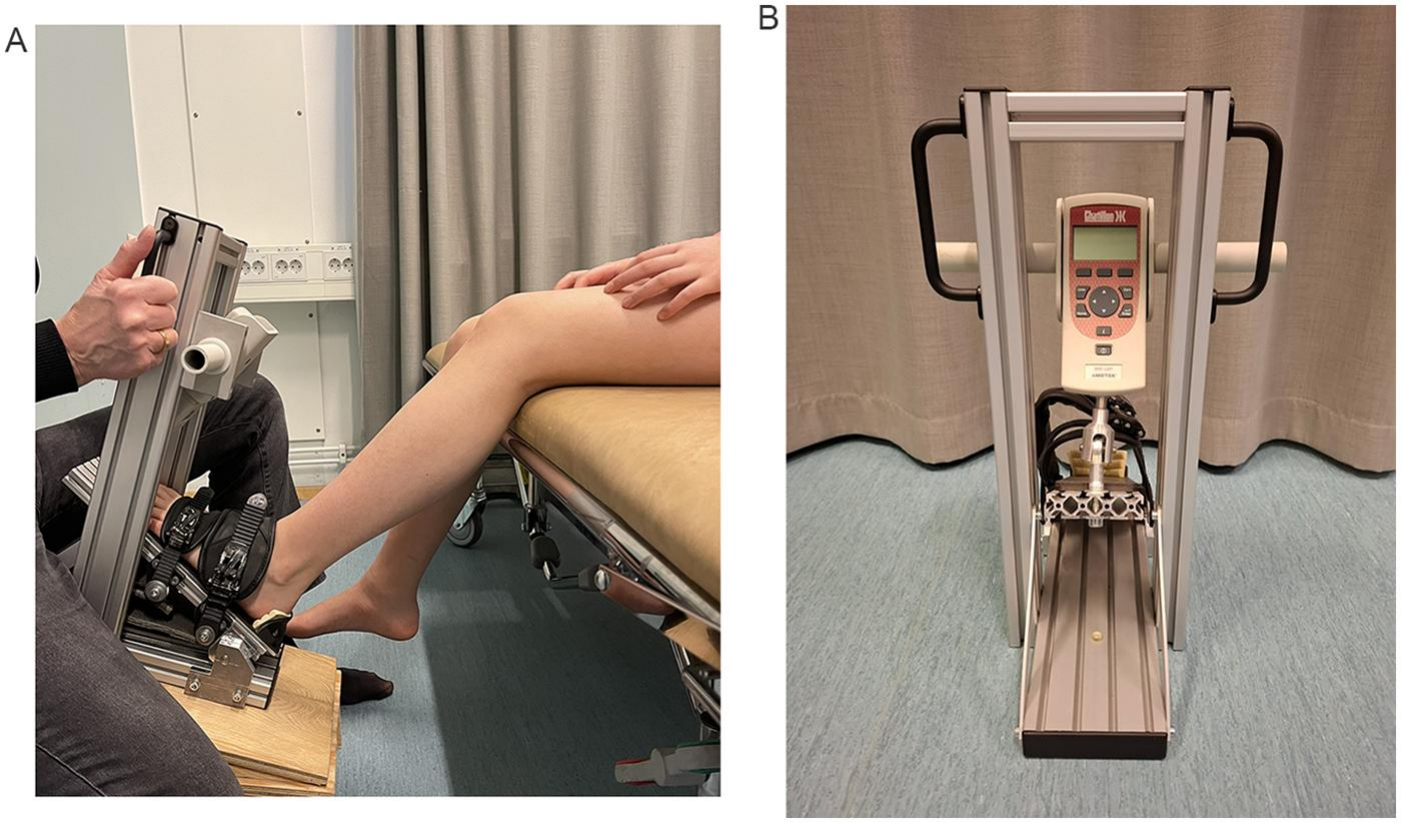
**(A)** force measurement using a rig-fixed dynamometer in a sagittal plane showing stabilization of the measurement apparatus, **(B)** the rig-fixed dynamometer in a frontal view.

Reliable quantification of both passive joint resistance and active muscle strength is essential for understanding neuromuscular development. While the NeuroFlexor captures the muscles response during passive movement, HHD-based voluntary strength measurements reflect the muscle's active functional capacity. By integrating these complementary assessment approaches, a more comprehensive understanding of pediatric muscle tissue, contractile behavior and motor function can be achieved. This approach is particularly relevant in children, given the pronounced developmental variability in neuromuscular properties and the clinical importance of reliable, age-appropriate assessment tools. One of the objectives of this study was therefore to evaluate the reliability of the NeuroFlexor for quantifying ankle joint hyper-resistance in typically developing children. The second objective was to evaluate the reliability of *a rig-fixed* dynamometer designed for measuring maximal voluntary isometric force of both plantarflexors and dorsiflexors of the ankle in children, see Figure 1.

## 2 Material and methods

Sample size followed Bujang and Baharum (20) guidelines recommending at least ten participants for acceptable precision in Intraclass Correlation Coefficient (ICC) estimation (20). Eleven conveniently recruited children aged 7–14 years: eight girls and three boys with a mean (SD) age of 10.1 (2.5) years, weight of 39.2 (11.4) kg and height: 144.8 (15.6) cm with no history of orthopedic injuries or neuromotor disorder participated. Written informed consent was obtained from all parents or legal guardians, and all children provided assent. The study adhered to the Declaration of Helsinki. Ethical approval was obtained from the Swedish Ethical Review Authority (DNR: 2014-1829-31-4, with addendums: 2016-286-32, 2018-2128-32, and 2022-0212-02) and the study was registered at ClinicalTrials.gov (NCT05447299).

### 2.1 Data acquisition

This study employed a within-subject test-retest design. Both assessments were conducted on the same day, at Karolinska University Hospital, Solna, Stockholm, Sweden, with a 2-hour interval between sessions. Each session began with a physical assessment, followed by the NeuroFlexor measurements and subsequently strength measurements using the *rig-fixed* dynamometer (HHD). All but two children completed bilateral assessments: one reported leg pain, the other had difficulties maintaining focus. Legs were examined in random order. To avoid violation of statistical independence due to within-subject correlation, only one leg per participant was included in the statistical analyses: the initiating leg was selected according to a predefined rule.

### 2.2 The NeuroFlexor and component quantification

Slow movement (5°/s) provides an estimate of the non-neural resistance components, whereas the resistance during faster movement (240°/s) yields an estimate of the neural contribution in the calf muscles during passive ankle plantarflexion (2). The non-neural components of passive joint resistance are primarily related to the intrinsic mechanical properties of the muscle-tendon unit and joint structures (21). Using biomechanical modelling, parameters such as elasticity (stiffness), viscosity, and non-linear components of the passive resistant torque can be estimated from the slow movement, while the neural contribution can be derived from the faster movement (7, 22, 23).

To accommodate children, the footplate and calf-supporter of the original NeuroFlexor were customized. Each child sat with back support, with 90° hip flexion, 30° knee flexion and the ankle in neutral position. One foot was tested at a time while the other rested on the floor. The child was encouraged to remain as relaxed as possible throughout the assessment. A detailed illustration of the NeuroFlexor setup has been published previously (24). The passive ankle range of motion during measurement ranged over 40° excursion, from maximal plantarflexion to individuals' maximal dorsiflexion, at slow and fast velocities. For each participant, ten slow and five fast movements were recorded. Three representative slow trials and three fast trials were selected, and the mean of each was used for parameter identification, in accordance with previously described methods (7).

The parameter identification procedure consisted of two steps and based on the assumption that the resistance measured during slow movement arose purely from non-neural components, whereas the resistance measured during fast movement represented the combined effects of both non-neural and neural components.

The parameter identification method of step one (slow movement) followed our previous study (24). Briefly, a non-linear least squares method was applied to individual slow movement trials to estimate four non-neural parameters: passive stiffness coefficient (*k_p,_*), viscosity coefficient (*B_p_*), and two non-linear components (*k*_1_, and *k*_2_) as defined in Equation 1.

$$T_{\mathrm{slow}\;}=k_{p}\cdot (\theta -\theta_{0})+B_{p}\cdot \dot{\theta }+k_{1}\exp(k_{2}\cdot (\theta -\theta_{0}))+T_{0}$$ (1)

where *T*_slow_ is the total joint resistance, *k_p_* is stiffness coefficient, θ is the joint angle, $\theta_{0}$ is the initial joint angle, *B_p_* is the viscosity coefficient, $\dot{\theta }$ is the angular velocity, *k_1_* and *k_2_* are non-linear coefficients, and $T_{0}$ is the offset resistance.

**Figure 2:**
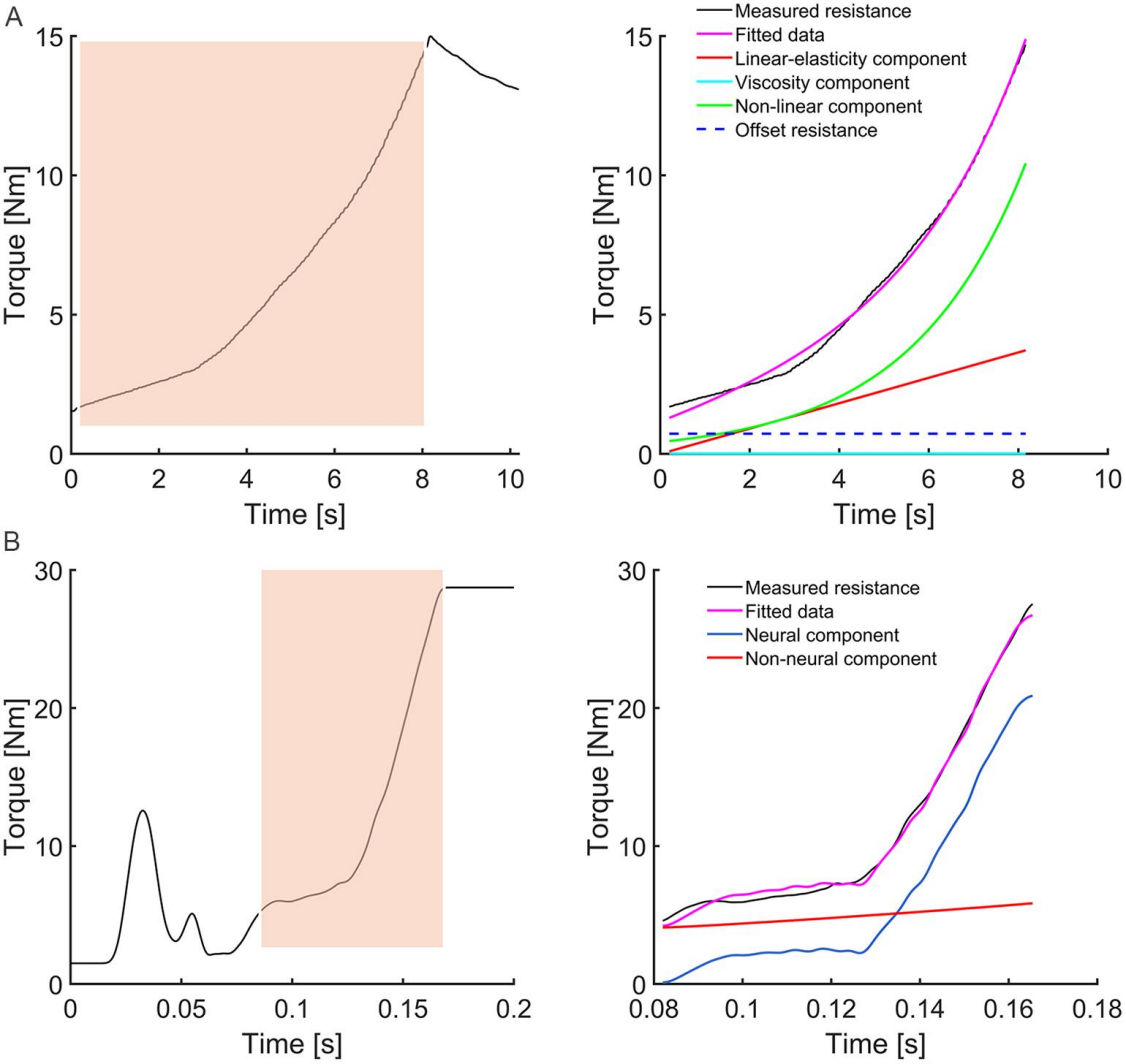
**(A)** Raw data of the measured joint resistance during one slow movement trial. Fitted data and with corresponding components of the raw torque. Fitting was performed only within the highlighted pink region. **(B)** Raw data of the measured joint resistance during one fast movement trial. Optimization was performed exclusively within the pink-shaded region, which corresponds to the steady-velocity phase of the fast movement, with acceleration and deceleration phases excluded. **(B)** Fitted data and with corresponding components of the raw torque.

In this equation, $k_{p}\cdot (\theta -\theta_{0})$ represents the linear-elasticity component, $B_{p}\cdot \dot{\theta }$ represents the viscosity component, and $k_{1}\exp(k_{2}\cdot (\theta -\theta_{0}))$ represents the non-linear component, see Figure 2A. Parameters estimates from each slow trial were averaged and subsequently used in the second step.

In the second step, the neural torque component (*T*_neu_) was assumed to be the residual torque by subtracting the non-neural component (*T*_non−neu_) from total measured resistance (*T*_fast_) during the fast movement, as shown in Equation 2.

$$T_{\mathrm{fast}\;}-T_{\mathrm{non}\text{-}\mathrm{neu}\;}=T_{\mathrm{neu}\;}$$ (2)

Where *T*_non_neu_ was computed using non-neural parameters identified in step one. *T*_neu_ was modelled according to Equation (3), which was derived from a modified torque-angle-velocity relationship describing joint torque as a function of joint angle and angular velocity, as established in a previous study (25). In this equation, *T_M_* represents the maximum joint torque of the *T*_neu_, and *y_1—_y_6_* are fitting parameters estimated using a non-linear squares approach. Optimization was performed in the steady-velocity phase of the fast movement, with acceleration and deceleration phases excluded, see Figure 2B.

$$T_{\mathrm{neu}\;}=T_{M}\left(\frac{y_{1}+y_{2}\dot{\theta }}{y_{3}+y_{4}\dot{\theta }}\right)\cdot \cos(y_{5}\theta +y_{6})$$ (3)

For further analysis, passive elasticity ($K_{p}$) and maximum joint torque of the neural torque component *(T_M_)* were selected, as these parameters are physiologically meaningful and primary contributors to the measured components of joint resistance. For simplicity, $K_{p}$ is referred to as the non-neural component, and *T_M_* as the neural component in the following sections.

Model performance was quantified using the variance accounted for (VAF), which reflects how well the model predicted torque values fit the measured data. VAF of the predicted torque was calculated as:

$$\mathrm{VAF}=1-\frac{\sum {\left(T_{\mathrm{fast}}^{m}-T_{\mathrm{fast}}^{e}\;\right)}^{2}}{\sum T{{}_{\mathrm{fast}}^{m}}^{2}}$$ (4)

where $T_{\mathrm{fast}}^{m}$ represents the experimental data and the $T_{\mathrm{fast}}^{e}$ model prediction. A VAF value of 1 indicates a perfect fit, whereas lower values reflect increasing deviation between measured and predicted torque Equation 4.

### 2.3 The HHD measurements

A hand-held dynamometer (Chatillion®, USA) was mounted on a custom-designed aluminum frame with handles and used for measuring maximal isometric strength, using a make-test technique, in which participants gradually build up force against a rigid surface over 4–5 s (26). A familiarization session consisting of one to two practice trials was conducted prior to data collection, followed by three maximal trials for each muscle group, starting with the plantarflexors. Rest intervals between trials were individualized based on each child's perceived readiness and typically ranged from 40 to 60 s to minimize fatigue. The rig featured an adjustable heel support, allowing precise alignment with ankle joint's axis of rotation when measuring maximal voluntary contraction torque for both plantarflexors and dorsiflexors. To accommodate children of varying sizes and ensure accurate alignment with individual joint axes, the rig-fixed HHD was placed on a height-adjustable inclined box. During testing, participants were seated on a height-adjustable examination bench without back-support, with 90° hip flexion, 30° knee flexion and a neutral ankle (0°). The thigh was supported by the bench, and participants were instructed to keep their trunk upright and hands resting on their laps (See Figure 1). The foot and lower leg were secured within the rig, while the HHD was stabilized by the examiner firmly holding the handles, minimizing movement and potential compensatory contributions from proximal joints (See Figure 1). Participants were verbally instructed and visually monitored throughout testing to avoid compensatory movements from the trunk, hip or knee. Torque was calculated by multiplying the force recorded by the HHD by the perpendicular distance between ankle joints axis and the lever arm, and the mean torque across three trials was used for analysis (27).

To date, normalized plantarflexor torque have not been published for children older than nine years (16), and previous pediatric studies have reported unnormalized isometric strength values, limiting comparability across individuals and studies (17). As normalization is essential in pediatric populations to discern whether observed strength increases reflect muscular development due to growth, averaged torque values were normalized to body weight and expressed as Nm/kg.

### 2.4 Statistical analysis

The ICC and corresponding 95% confidence intervals were calculated using IBM SPSS Statistics version 27.0 (IBM Corp., Armonk, NY, USA) based on a mean-rating (*k* = 3), absolute-agreement, two-way mixed-effects model. ICC values were interpreted according to Koo & Li et al. (28) as follows: <0.5, poor; 0.5–0.75, moderate; 0.75–0.9, good; and >0.90, excellent reliability. The standard error of measurement (SEM) was calculated as SEM $=\mathrm{standard}\;\mathrm{deviation}\;(\mathrm{SD})\times \sqrt{\left(1-\;ICC\right)}\;$ and the Minimal detectable change (MDC) was calculated as MDC =SEM∗1.96∗√2 (23). Agreement between *Test* A and *Retest* B was assessed using Bland-Altman plots, where the difference (*Retest* B—*Test* A) was plotted against their mean for each participant. A sensitivity analysis was performed to assess the impact of outliers on the results, with analyses performed both including and excluding the outlier.

## 3 Results

### 3.1 The NeuroFlexor

**Figure 3:**
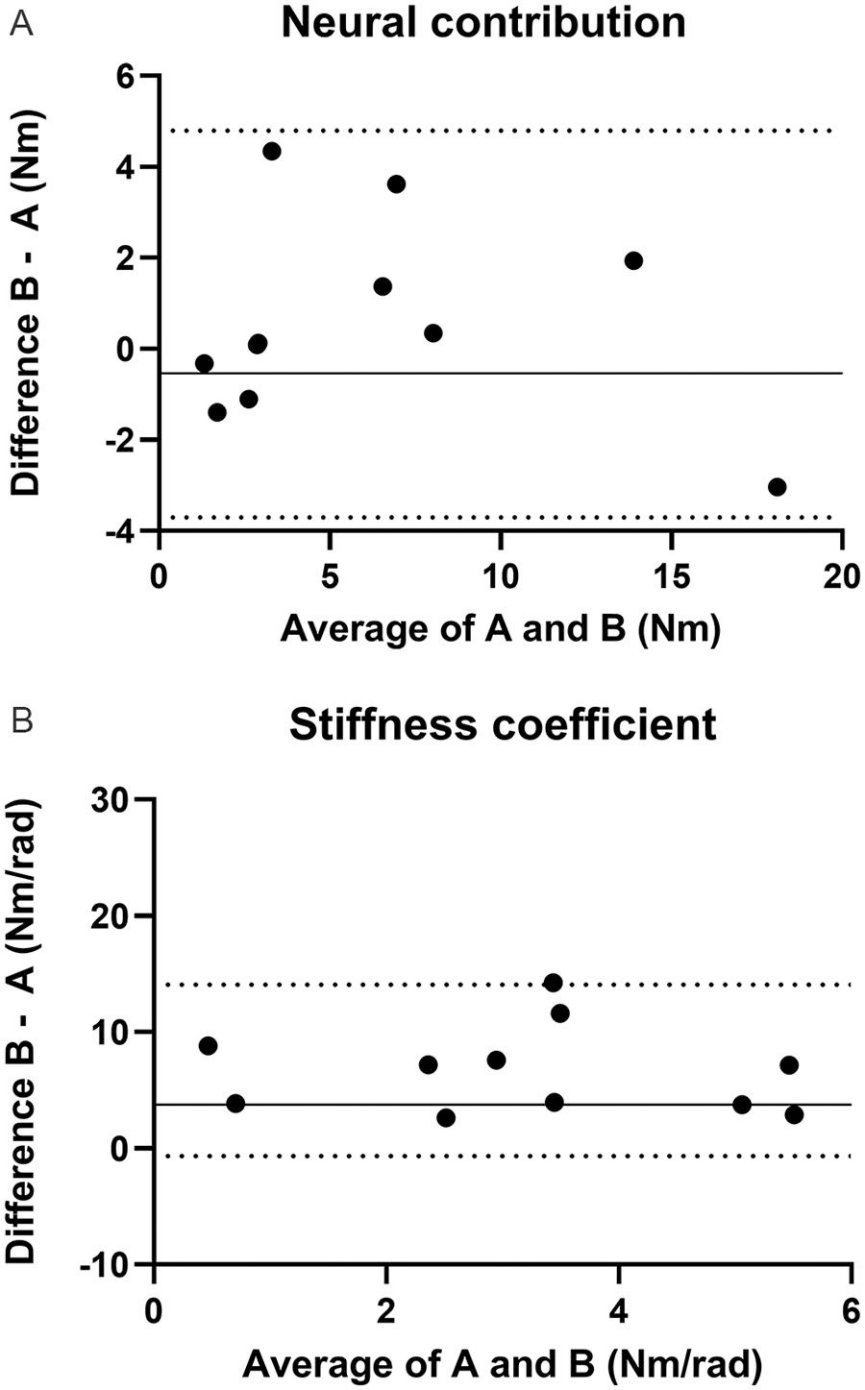
Bland-Altman plot illustrating the agreement between *test* (A) and *retest* (B) for the NeuroFlexor assessments. The *x*-axis represents the average of the two examinations, while the *y*-axis displays the difference between the test-retest examinations (B-A). The line indicates the mean differences, and the dotted lines indicate the Limits of Agreement (LoA) for **(A)** Estimated neural contribution to passive joint resistance (*T_M_*) with a bias of −0.54 Nm. Upper LoA: 3.71 Nm, Lower LoA: −4.80 Nm. **(B)** Estimated stiffness coefficient ($K_{p}$) from the NeuroFlexor with a bias of 3.77 Nm/rad, Upper LoA: 14.1 Nm/rad, Lower LoA: −0.68 Nm/rad. All data points lie within these limits, indicating good agreement between the two examinations.

The assessment of passive joint resistance with the NeuroFlexor demonstrated excellent test-retest reliability for the neural component, *T_M_*: *Test*, mean (SD) 6.47 (5.33) Nm vs. *Retest*, 5.97 (5.69) Nm resulting in an ICC of 0.96 (95% CI: 0.86, 0.99) (see Figure 3A). The non-neural component (stiffness coefficient) showed good reliability, $K_{p}$: *Test* 6.87 (4.05) Nm/rad vs. *Retest*, 6.97 (2.96) Nm/rad, with an ICC of 0.78 (95% CI: 0.13, 0.94) (see Figure 3B). The modelled neural torque closely matched the measured ankle resistance; VAF *Test*: 0.998, VAF *Retest*: 0.997. NeuroFlexor components showed good agreement on the Bland-Altman plot.

### 3.2 The HHD

**Figure 4:**
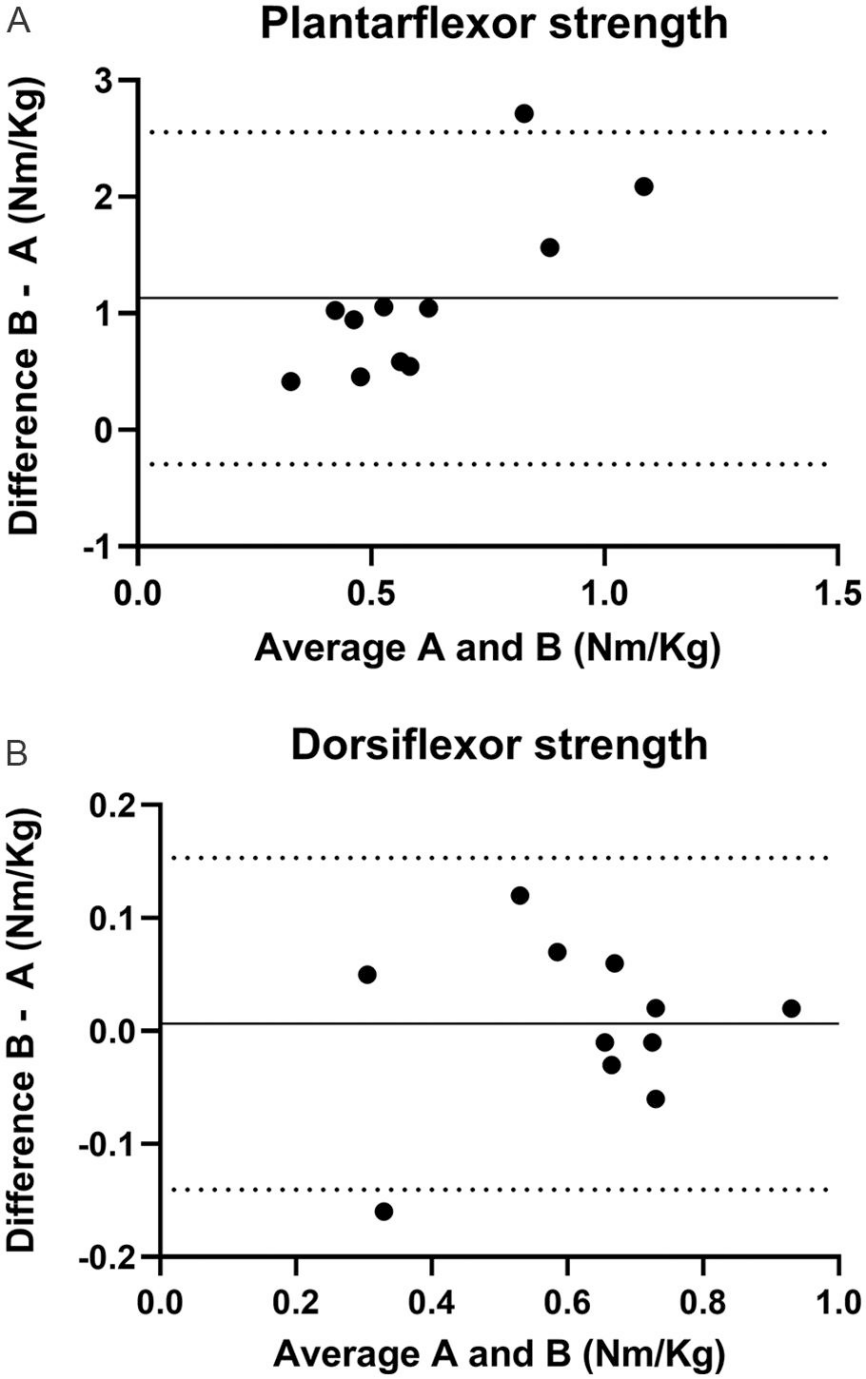
Bland-Altman plot illustrating the agreement between *test* (A) and *retest* (B) for the NeuroFlexor assessments. The *x*-axis represents the average of the two examinations, while the *y*-axis displays the difference between the test-retest examinations (B-A). The line indicates the mean differences, and the dotted lines indicate the Limits of Agreement (LoA) for **(A)** Plantarflexor strength with a bias of 1.13 Nm/kg. Upper LoA: 2.56 Nm/Kg, Lower LoA: −0.29 Nm/Kg. **(B)** Dorsiflexor strength with a bias of 0.006. Upper LoA: 0.15 Nm/Kg, Lower LoA: −0.14 Nm/Kg. Most data points lie within these limits, indicating good agreement between the two examinations.

Muscle strength measurements for the plantarflexors and dorsiflexors revealed excellent test-retest reliability. Plantarflexor strength at *Test* mean (SD) 1.16 (0.64) Nm/kg vs. *Retest*, 1.21 (0.50) Nm/kg resulting in an ICC of 0.96 (95% CI: 0.86, 0.99) (see Figure 4A). Excluding the outlier resulted in a bias of 1.035 (compared with 1.131 when included) and limits of agreement (LoA) ranging from −0.07 to 2.01 (vs. −0.29 to 2.56), indicating that the outlier had little effect on the results. Dorsiflexor strength at *Test* was 0.62 (0.18) Nm/kg vs. *Retest,* 0.63 (0.19) Nm/kg at, resulting in an ICC of 0.96 (95% CI: 0.86, 0.99) (see Figure 4B). Excluding the outlier reduced the bias from 0.07 to 0.053 and narrowed the LoA from −0.14 to 0.153, and −0.08 to 0.128, showing minimal impact on the results.

The two strength measurements lay outside the 95% LoA on the Bland-Altman plot. One strength observation exceeded the upper limit, consistent with the expected ∼5% of values outside these bounds, one observation fell short of the lower LoA, (see Figures 4a,B). Data including SEM and MDC values are summarized in Table 1.

**Table 1:** Intraclass correlation coefficient values for the NeuroFlexor measurement and strength measurements by the rig-fixed HHD.

Outcome measure	A mean (SD)	B mean (SD)	ICC	95% CI	SEM	MDC
Elasticity/Stiffness coefficient, *K_p_* Nm/rad	6.87 (4.05)	6.97 (2.96)	0.78	0.13, 0.94	1.24	3.43
*T_M_* Nm	6.47 (5.33)	5.97 (5.69)	0.96	0.86, 0.99	1.02	2.84
Averaged MVC PF Nm/Kg	1.16 (0.64)	1.21 (0.50)	0.96	0.86, 0.99	0.11	0.30
Average MVC DF Nm/Kg	0.62 (0.18)	0.63 (0.19)	0.96	0.86, 0.99	0.03	0.10

Variables from the NeuroFlexor (NF): Elasticity/Stiffness $K_{p}$ coefficient in the lower limb obtained from the slower motion, and the $T_{M}$: The maximal neural torque fast NF measurement. SEM: standard error of measurement. MDC: Minimal detectable change. Averaged isometric strength values of Maximal isometric voluntary contraction (MVC) obtained by the rig-fixed HHD: in PF: Plantarflexor, and DF: Dorsiflexors.

## 4 Discussion

Our study aimed to evaluate the test-retest reliability of two instruments for assessing neuromuscular function in children: the NeuroFlexor for passive resistance and a rig-fixed dynamometer for voluntary muscle strength. Both instruments showed strong and reliable psychometric properties in typically developing children aged 7–14 years, supporting their use as reliable tools for the quantitative assessment of neuromuscular development.

The NeuroFlexor showed good to excellent test-retest reliability for measuring passive joint resistance in both the non-neural, and the neural component. To our knowledge, this is the first study to report reliable measurements using this instrument in a pediatric population. Previous studies in adult post-stroke assessing the ankle joint reported similar results, showing excellent reliability for the neural component, with ICC values of ≥0.90, supporting its utility in adult populations (9). Comparable reliability using similar methodology has also been reported in the wrist joint in adult post-stroke (10).

We have previously used the same NeuroFlexor setup in children with cerebral palsy (CP), where the testing procedure proved feasible and well tolerated, and completed by all participants without adverse advents (24). In the present study, the non-neural component of joint resistance was characterized by the stiffness coefficient as it represents the dominant contributor. This approach is consistent with our previous work (24), in which stiffness coefficient (*k_p,_*), was the primary driver of between-group differences, while the viscosity coefficient (*B_p_*), and two non-linear components (*k_1_*, and *k_2_*) contributed minimally and showed no significant group differences (24).

In the present study, the excellent reliability observed for measuring the neural component indicates promising potential for the NeuroFlexor as a feasible and consistent tool for assessing passive joint resistance in pediatric populations. Using our current setup, we also demonstrated that combining biomechanical modeling **with** a motorized device can reliably distinguish neural from non-neural contributions to ankle joint resistance during passive dorsiflexion. This distinction is clinically important, as quantifying the relative contributions of neural vs. non-neural resistance may support more informed clinical assessment and targeted treatment planning. By providing objective information about underlying resistance components, such measurements may help clinicians better define individual impairments profiles and guide both the selection and evaluation of interventions. Moreover, the relationship between muscle strength and passive resistance of the plantarflexors remains insufficiently explored, and objective characterization of these interacting components may enhance the interpretation of functional limitations and treatment effects. In this context, the reported MDC values provide reference estimates for interpreting change at the individual level by indicating whether observed changes exceed measurement error, particularly given the absence of previously reported MDC data for this assessment in pediatric populations (27). Such mechanistic insights may also contribute to future research aimed at improving therapeutic strategies for children with neurological impairments.

The rig-fixed HHD showed excellent test-retest reliability for both plantarflexion and dorsiflexion strength measurements, contrasting with earlier pediatric studies in which lower-limb strength assessments using HHD were limited by methodological variability, reduced validity, or insufficient reliability, particularly for the plantarflexors (29–32). The ankle was tested in neutral position, a commonly used configuration in plantarflexor strength testing to optimize force production and ensure methodological consistency (33, 34). While weight-bearing, force-plate-based assessments may offer higher ecological validity for certain functional tasks, such approaches typically require specialized equipment and controlled laboratory conditions, which may limit feasibility in many pediatric clinical settings (35). Using this rig-fixed setup, we demonstrated that muscle strength testing is both feasible and reliable in children aged 7–14 years in a clinical environment, with no child reporting discomfort or displaying behavioral signs of distress during testing, suggesting that participant discomfort did not influence data quality. Moreover, the fixed dynamometer setup minimizes the confounding influence of examiner strength, enabling more accurate assessment of the child's muscle performance and overcome previously reported difficulties in testing children older than nine years (16).

One key methodological consideration is how best to normalize strength values across children of different ages, an issue receiving increasing attention. Although no universal consensus exists, muscle strength is commonly reported as raw force (Newtons) or torque (Newton Meters), and as normalized measures, typically relative to body mass, depending on the study design (16, 17, 36, 37). In this study, we normalized muscle strength to body mass (Nm/kg) using a ratio scaling approach, which assumes a linear relationship between strength and body size. While allometric scaling may better account for size-related variance in pediatric populations (38), ratio scaling was chosen for its simplicity and wide use in the literature, thereby facilitating comparison across studies. Establishing reliable, normalized strength values is particularly relevant for quantifying motor impairments and evaluating intervention outcomes in populations such as children with CP. The influence of muscle strength on functional capacity remains ambiguous, particularly in children with motor disorders such as cerebral palsy (CP), who often present difficulties in voluntarily producing maximal force during isolated testing. For example, stronger plantarflexors assessed using handheld dynamometry (HHD) have been reported in higher functioning children with spastic CP (GMFCS I) compared with those classified as GMFCS II and III (37). However, higher plantarflexor forces have also been demonstrated during gait compared with seated testing using instrumented measurements (39) suggesting that functional force generation during walking may exceed values obtained with HHD. This discrepancy supports the view that activity limitations in CP may be strongly influenced by task-specific, activity-based factors than by individual motor impairments alone (40). Accordingly, muscle strength may contribute to functional walking capacity indirectly and in interaction with factors such as motor control, coordination, and task demands, rather than serving as a sole determinant of gait performance. In this context, muscle strength reflects voluntary force-generating capacity, whereas resistance to passive movement reflects involuntary neural and non-neural mechanisms. Together, they provide complementary information about neuromuscular impairment. Although the present study does not establish normative values, it provides preliminary evidence for the feasibility of our instrument and its potential utility in developing standardized strength profiles across pediatric age groups. However, our relatively small sample size underlines the need for larger cohorts before normative reference values can be established. Future studies should also explore what magnitude of change is clinically meaningful difference at the individual level for NeuroFlexor measurement, particularly in clinical or longitudinal settings.

Several limitations should be considered when interpreting the present findings. The relatively small sample size may limit generalizability and contribute to wider confidence intervals in some reliability estimates. Test-retest assessments were conducted on the same day with a short inter-session interval, which may have introduced reduced biological variability or learning effects, potentially inflating reliability. However, this design was chosen to minimize developmental and environmental variability in this pediatric cohort. Despite standardized positioning and joint stabilization, subtle compensatory movements at the trunk or proximal joints cannot be entirely excluded during maximal efforts. Examiner-related influences may also have contributed to measurement variability. In addition, the inclusion of both sexes and a relatively wide age range may have introduced heterogeneity related to growth and maturation. Physical activity levels were not formally assessed, which could influence muscle strength and neuromuscular outcomes. Despite these limitations, the findings provide important insight into short-term test-retest reliability of instrumented ankle resistance and strength measurements in children.

In conclusion, instrumented assessments of passive joint resistance using the NeuroFlexor demonstrated good to excellent test-retest reliability for quantifying neural and non-neural components in typically developing children. Additionally, isometric ankle strength measurements acquired using a rig-fixed HHD showed likewise excellent test-retest reliability. These findings support the reliability of both instruments for assessing ankle resistance and strength in pediatric populations. Future research should focus on establishing normalized strength values, clinically meaningful threshold for change—particularly in children with motor impairments, and to further evaluate the feasibility and applicability of these instruments in longitudinal and intervention-based studies.

## Data Availability

The raw data supporting the conclusion of this article will be made availible upon request by the authors, without undue reservations.
