# Supplementary material for: Good to excellent reliability of instrumented ankle passive resistance and strength measurements in children
Source: Front Pediatr. 2026 May 28;14:1832116. doi: 10.3389/fped.2026.1832116 (PMC13254162; doi:10.3389/fped.2026.1832116)
Supplement: Supplementary file 1 [file Table1.docx]

| Age | Weight | Height | A PF N | B PF N | A DF N | B DF N |
| --- | --- | --- | --- | --- | --- | --- |
| 7 | 23.4 | 122 | 222 | 230 | 42 | 30 |
| 7 | 32.9 | 140 | 241 | 260 | 106 | 110 |
| 8 | 24.8 | 128 | 89 | 111 | 67 | 67 |
| 9 | 48.6 | 137 | 142 | 183 | 63 | 83 |
| 9 | 34.4 | 140 | 176 | 235 | 227 | 112 |
| 9 | 32.4 | 141 | 171 | 161 | 106 | 107 |
| 10 | 44.9 | 138 | 250 | 259 | 97 | 102 |
| 12 | 44.3 | 156 | 194 | 219 | 183 | 179 |
| 12 | 38.1 | 151 | 361 | 404 | 126 | 117 |
| 13 | 62.8 | 171 | 137 | 173 | 182 | 191 |
| 15 | 44.3 | 170 | 153 | 202 | 95 | 126 |

# Supplementary Material

[Supplementary table of raw muscle strength values in Newton]

Table. S1.

| Age | Weight | Height | A PF N | B PF N | A DF N | B DF N |
| --- | --- | --- | --- | --- | --- | --- |
| 7 | 23.4 | 122 | 222 | 230 | 42 | 30 |
| 7 | 32.9 | 140 | 241 | 260 | 106 | 110 |
| 8 | 24.8 | 128 | 89 | 111 | 67 | 67 |
| 9 | 48.6 | 137 | 142 | 183 | 63 | 83 |
| 9 | 34.4 | 140 | 176 | 235 | 227 | 112 |
| 9 | 32.4 | 141 | 171 | 161 | 106 | 107 |
| 10 | 44.9 | 138 | 250 | 259 | 97 | 102 |
| 12 | 44.3 | 156 | 194 | 219 | 183 | 179 |
| 12 | 38.1 | 151 | 361 | 404 | 126 | 117 |
| 13 | 62.8 | 171 | 137 | 173 | 182 | 191 |
| 15 | 44.3 | 170 | 153 | 202 | 95 | 126 |

Abbreviation: Test occasion A, PF Plantar flexor, N Newton, B Test occasion B, DF Dorsiflexors. Weight in kilograms and height in centimetres.
